# The Effects of Family Sports on Mental Health in Children and Adolescents: A Meta-Analysis

**DOI:** 10.3390/bs16050776

**Published:** 2026-05-14

**Authors:** Shaofeng Peng, Chuangtao Li, Jingsong Wang, Shen Wang

**Affiliations:** School of Physical Education and Sport Science, Fujian Normal University, Fuzhou 350117, China

**Keywords:** family physical activity, mental health, children and adolescents, effectiveness of interventions, meta-analysis

## Abstract

**Objective**: To systematically review the effects of family-based physical activity interventions on the mental health of children and adolescents and identify potential moderators. **Methods**: Following PRISMA guidelines, we searched PubMed, PsycARTICLES, PsycINFO, Elsevier, Web of Science, Cochrane, and three major Chinese databases for randomized controlled trials and non-randomized controlled trials on family-based physical activity interventions targeting mental health in children and adolescents aged 5–19 years. Searches were conducted through 10 February 2026. Two reviewers independently screened studies, extracted data, and assessed risk of bias. Meta-analyses were performed using CMA 3.3. **Results**: Eleven studies involving 1160 participants were included. The random-effects model indicated that the overall pooled effect size for family physical activity interventions included in this study was (g = 0.443, 95% CI: 0.272–0.614), suggesting that family physical activity interventions, regardless of their specific components, are generally associated with improved mental health in children and adolescents. Further analysis revealed that this overall effect reached statistical significance in the positive mental health dimensions (e.g., self-esteem, emotional well-being) (g = 0.467, 95% CI: 0.271–0.663), whereas it did not reach statistical significance in the negative psychological symptoms dimensions (e.g., depression) (g = 0.358, *p* > 0.05). Subgroup analyses indicated that intervention location (home-based group g = 0.26 vs. non-home-based group g = 0.55), intervention duration (≤3 months, g = 0.54 vs. >3 months, g = 0.36), and program type (non-multicomponent programs, g = 0.26 vs. multicomponent programs, g = 0.55) showed no statistically significant differences in their effects across groups (*p* > 0.05). No significant effects were observed in the overall meta-regression model. **Conclusions**: Current evidence suggests that family involvement may provide a more conducive setting for physical activity interventions targeting children and adolescents; overall, such interventions are associated with improvements in positive mental health. However, this finding should be interpreted as a composite estimate of intervention programs across various heterogeneous factors, such as different modes of family involvement and program components, and their effects on reducing negative psychological symptoms remain unclear. Future research should further refine the composition of these interventions and conduct high-quality, long-term studies to clarify their key components and long-term effects.

## 1. Introduction

Mental health issues affect 10% to 20% of children and adolescents worldwide and account for a significant portion of the global disease burden, with detection rates on the rise (The, 2017, 2022). To address this trend, numerous studies have explored non-pharmacological, easily scalable intervention approaches, and the positive effects of physical activity have been systematically validated by multiple meta-analyses (Firth et al., 2020; Kieling et al., 2011). The two-factor model suggests that mental health comprises two interrelated yet relatively independent dimensions: positive mental health, characterized by self-esteem, life satisfaction, and well-being; and negative mental symptoms, represented by depression, anxiety, and stress. Defining mental health solely by the reduction of symptoms risks overlooking differences in individuals’ levels of positive adaptation (Magalhaes, 2024). Existing evidence also indicates that physical activity is not only associated with the alleviation of negative psychological symptoms but also with improvements in self-esteem, social competence, and overall mental health levels (Ahn & Fedewa, 2011; Beauchamp et al., 2018; Q. Fu et al., 2025; Singh et al., 2026; Rao & Shi, 2025). However, these benefits are not determined solely by individual activity behaviors; mental health is also jointly shaped by social contexts and early developmental environments (Kieling et al., 2011; Tao, 2021). The Sociology of Mental Health perspective posits that mental health is the result of the interaction between humans and their environment, with living conditions and social structures serving as the fundamental determinants of mental health (Yu & Wang, 2026). From the perspective of the social ecological model, the family serves as a key microsystem linking behavioral formation, emotional experiences, and psychological development (Hu et al., 2021; Su et al., 2022). Parental support, family companionship, and shared participation not only influence children’s and adolescents’ levels of physical activity but may also further impact their psychological adjustment through relational bonds, role modeling, and daily support (Brennan et al., 2025; Rhodes et al., 2024; Yang et al., 2025).

Given the foundational role of the family in the psychological development of children and adolescents, family-based physical activity interventions have gradually emerged as an important non-pharmacological approach to addressing mental health issues among adolescents. Although existing research does not yet provide a completely consistent definition of such interventions, the core consensus lies in viewing the family as a crucial unit through which the intervention exerts its effects (Brennan et al., 2025; Rhodes et al., 2024; Su et al., 2022; Wang et al., 2025). In terms of the specific forms of family members’ participation in children’s and adolescents’ physical activities, family-based physical activity interventions primarily take two forms. First, family members directly participate in physical activities together (Paudel et al., 2025; Uijtdewilligen et al., 2017). The significance of this approach lies not in having children and adolescents complete predetermined exercise tasks under family supervision, but rather in the family cohesion, improved parent–child relationships, and subsequent engagement in physical activity that result from shared participation. These factors further translate into more robust emotional support and more positive psychological experiences, thereby promoting the mental health of children and adolescents—a benefit that is particularly evident among children in early to middle school age (Feng & Xu, 2025; Ornelas et al., 2007; Rhodes et al., 2024). Parents or other caregivers provide assistance and support through encouragement, modeling, accompaniment, rule-setting, goal-setting, and process monitoring (Rhodes et al., 2010, 2020a, 2020b; Yao & Rhodes, 2015). Among these, encouragement, modeling, and accompaniment help enhance children and adolescents’ positive expectations and willingness to participate in physical activity. On the other hand, rule-setting, goal-setting, and process monitoring provide them with a clearer behavioral framework and external constraints, facilitating the initiation and maintenance of physical activity (Y. Jiang & Xiao, 2024; Rhodes et al., 2010, 2020b; Yao & Rhodes, 2015); Compared to simple modeling, comprehensive support that includes emotional support and behavioral guidance is more effective in helping children and adolescents develop sustained participation experiences and a stable sense of control, which may further translate into positive mental health benefits (Brennan et al., 2025; Hong et al., 2020; Rhodes et al., 2010, 2020b; Su et al., 2022; Yao & Rhodes, 2015). It should be noted that in practice, family physical activity interventions rarely take a single form; some programs integrate additional components such as dietary guidance, behavior management, or lifestyle adjustments on top of physical activity as the core component, making the pathways through which they influence the mental health of children and adolescents more complex (Enright et al., 2020; Fernández-Lázaro et al., 2024; Wills-Ibarra et al., 2024). Consequently, differences in mental health outcomes across studies may reflect not only variations in intervention intensity or duration but also differences in program structure and implementation conditions. Accordingly, the family-based physical activity interventions discussed in this paper do not refer broadly to all family-related health promotion activities, but rather to intervention programs that primarily target children and adolescents, are oriented toward promoting their mental health, center on physical activity as the core intervention component, and utilize the family as the key unit of implementation.

Based on the conceptual definitions outlined above, the impact of family physical activity interventions on the mental health of children and adolescents remains to be systematically clarified. While existing reviews have largely confirmed the psychological benefits of various physical activities and the significance of the family system for children and adolescents, systematic meta-analyses examining evidence-based research on the integration of family units and physical activities remain relatively limited. In particular, existing studies still exhibit differences in intervention design, implementation conditions, and outcome measures. Therefore, the actual effects of family physical activity interventions on the mental health of children and adolescents, as well as their varied manifestations, require further clarification. Based on this, the present study aims to explore the intervention effects of family physical activity interventions on the mental health of children and adolescents, as well as the key moderating variables, to provide targeted references and recommendations for optimizing future intervention designs and policy formulation.

## 2. Method

This study was conducted in strict accordance with the PRISMA statement criteria (Liberati et al., 2009) and the study protocol registered in PROSPERO (CRD420261328040).

### 2.1. Inclusion and Exclusion Criteria

The inclusion and exclusion criteria for the literature are as follows: Inclusion criteria: (1) Study participants must be children and adolescents aged 5–19 years; (2) Controlled trials, including randomized controlled trials and non-randomized controlled trials; control conditions are not limited to active or passive controls, but must include a comparable reference condition to assess changes in the mental health status of children and adolescents before and after the family physical activity intervention; (3) Intervention measures: physical activity interventions with the family as the key unit, involving at least one caregiver participating in physical activities alongside the adolescent (including participation, assistance, supervision, and support). The intervention content must primarily consist of physical activity (e.g., sports, games, outdoor activities), and the intervention must take place in the home environment or be family-led; (4) Outcome variables: The indicators must be measured using psychometrically validated mental health assessment tools, including established scales or researcher-developed instruments for which basic reliability and validity information has been reported in the original study. At least one mental health-related outcome must be reported, including but not limited to any of the following categories: Psychopathology dimension, involving negative psychological states such as mental disorders (e.g., depression, anxiety), psychological distress, and internalizing and externalizing symptoms; Positive mental health dimensions, such as life satisfaction, emotional well-being, psychological well-being, and social well-being, which represent positive psychological states and functioning.

Exclusion criteria: (1) Studies involving clinical patients with mental disorders or psychological illnesses, as interventions for patients with psychological illnesses are more complex and require separate studies (Xu et al., 2017); (2) Studies in which physical activity is not the core intervention but only an ancillary component; (3) Studies in which the family is not included as a key unit of implementation; (4) Studies in which caregivers provided only general information, passive cooperation, or logistical support; (5) Studies from which mental health-related outcome data could not be extracted, or in which outcome measures were not assessed using clearly defined measurement tools; (6) Literature not published in Chinese or English.

### 2.2. Literature Review

Relevant literature was retrieved from six foreign-language databases—PubMed, PsycARTICLES, PsycINFO, Elsevier, Web of Science, and Cochrane—as well as three Chinese databases: CNKI, Wanfang, and VIP. The search strategy was developed around four core concepts: physical activity or exercise, family or caregiver involvement, children and adolescents, and mental health outcomes. Controlled vocabulary terms, such as MeSH terms in PubMed, were combined with free-text terms using Boolean operators, and the strategy was adapted for each database. Corresponding Chinese search terms were used in the Chinese databases. The full database-specific search strategies are provided in the Supplementary Material. The initial search was conducted in January 2026, and the final literature search was completed on 10 February 2026. The literature was screened by the first author and verified by the second author; any disagreements were resolved through discussion with the corresponding author.

### 2.3. Data Analysis

A meta-analysis was conducted using Comprehensive Meta-Analysis Version 3.3.

#### 2.3.1. Data Extraction and Quality Assessment

We extracted and coded the characteristics of each study and the outcome data included in the analysis. Data extraction and coding were performed independently by two authors; in cases of disagreement, the final coding was determined after consultation with the corresponding author. The coding of study characteristics included: author (year), study type, sample size, measurement time points, participant type, age, intervention type, primary intervention site, intervention frequency/duration, intervention cycle, outcome variables, measurement tools, assessment results of measurement tools, and study quality assessment results. Specific coding is shown in Table 1.

Based on the Dual-Factor Model of mental health, the mental health data extracted in this study are divided into two categories (Magalhaes, 2024). The specific data extraction rules are as follows: (1) If a single study reports indicators from both dimensions, data from both categories are extracted; (2) If a single dimension includes multiple measurement indicators, priority is given to extracting the indicator with the higher effect size or higher prevalence in the study population; (3) For scale data, priority is given to extracting the total scale score; if only subscale scores are reported, the subscale score significantly correlated with the outcome variable is selected (Livingston & Boyd, 2010).

Family physical activities are categorized into two intervention types based on the level of caregiver involvement. Joint participation: Caregivers and children engage in the same or complementary physical activities simultaneously in the same space, creating direct physical interaction. Parents act as co-participants rather than merely supervisors or demonstrators (Paudel et al., 2025; Uijtdewilligen et al., 2017). Assistive support: Although caregivers do not participate in the physical activity in real time throughout the entire session, they are deeply involved in setting goals, monitoring the process, and providing feedback for the child’s physical activities. The primary roles of parents include supervision, assistance, support, and demonstration (Rhodes et al., 2010, 2020a, 2020b; Yao & Rhodes, 2015).

For multi-arm randomized controlled trials, this study first determined the criteria for combining intervention arms based on clinical and methodological homogeneity. If multiple homogeneous intervention groups share the same control group, to avoid unit of analysis error, this study followed the relevant methodological recommendations in the Cochrane Handbook and the guidelines by Axon et al. on the handling of multi-arm studies, combining homogeneous intervention groups that met the inclusion criteria into a single intervention group for comparison with the control group (Axon et al., 2023; Cumpston et al., 2019). For continuous outcomes, the sample-weighted pooled mean was calculated, and the standard deviation was computed using the formula in the handbook; for dichotomous outcomes, the effect size was calculated based on the pooled number of events and sample size.

Conduct separate quality assessments of the measurement tools and the studies. Given that the outcome measures in the included studies primarily rely on mental health scales, and that different measurement tools may vary in terms of reliability, validity, and validation for specific populations, we adopted the three risk categories established by Clement et al. (2013)—low risk, high risk, and unclear risk—to assess the quality of the measurement tools, thereby aiding in the description of the reliability of outcome measurement (Clement et al., 2013). Low risk: (1) Cronbach’s *α* ≥ 0.7, or references indicating that the measurement instrument is reliable or valid. High risk: (1) Measurement tools developed by the study authors themselves, with no psychometric data reported; (2) Cronbach’s *α* < 0.7; (3) Unreferenced measurements. Unclear risk: (1) References exist but psychometric data are not reported; (2) References exist but the measurement tool is not stated to be reliable or valid; (3) Validated measurement tools used for the first time in a different population. The Cochrane Risk of Bias tool version 1 (RoB 1) was used to assess the risk of bias of the included studies (Cumpston et al., 2019). It covers six domains—selection bias (including random sequence generation and allocation concealment), performance bias (including blinding of researchers and participants), detection bias (blinded assessment of study outcomes), attrition bias (completeness of outcome data), reporting bias (selective reporting of study results), and other sources of bias—totaling seven items. Each item was judged as “low risk of bias,” “unclear risk of bias,” or “high risk of bias,” and the overall risk-of-bias judgement for each study was summarized based on the domain-level assessments using the same terminology.

#### 2.3.2. Calculation of Effect Sizes

This study used the standardized mean difference, Hedges’ *g*—a modified version of Cohen’s *d*—as the effect size measure to more accurately estimate the effects of small-sample studies (Vollestad et al., 2012). Effect size calculations were performed using the CMA 3.3 software. The meta-analysis primarily pooled post-intervention follow-up data from the first measurement after the intervention ended, and calculated Hedges’ g directly based on the sample sizes, mean scores, and standard deviations of the intervention and control groups; if the original study did not report means or standard deviations, effect sizes were derived from statistical measures such as *χ*^2^, *t*, and *F* (Ren et al., 2020). Effect sizes are categorized as follows: 0.2 indicates a small effect, 0.5 a moderate effect, and 0.8 a large effect (Dunning et al., 2019).

Given that the scoring directions of the scales included in the study were not entirely consistent, this study standardized the direction of effect sizes prior to conducting a meta-analysis. For outcome measures where higher scores indicate better mental health, the original scoring direction was retained; for outcome measures where higher scores indicate more severe mental illness or poorer mental health, reverse coding was applied when calculating standardized mean differences (Ren et al., 2020). After this adjustment, all effect sizes were interpreted in a consistent manner: a positive g value (*g* > 0) indicates that the family physical activity intervention produced a beneficial effect, while a negative g value (*g* < 0) indicates that the intervention had no beneficial effect or produced an adverse effect.

Effect sizes were calculated using a random-effects model, and moderator analysis was conducted using a mixed-effects model (Berkeljon & Baldwin, 2009; Carrero et al., 2019). For data analysis, *Q* and *I*^2^ were used to assess heterogeneity. *I*^2^ represents the proportion of total variance attributable to between-study variance (*I*^2^ = 25%, 50%, and 75% indicate low, moderate, and high heterogeneity, respectively). When *Q* is significant and *I*^2^ ≥ 75%, this indicates the presence of substantial heterogeneity among studies, suggesting that the selection of a random-effects model is appropriate (Huedo-Medina et al., 2006).

#### 2.3.3. Assessment of Publication Bias and Sensitivity Analysis

We used funnel plots and the fail-safe number to conduct a preliminary assessment of the risk of publication bias (Khoury et al., 2013; Morgan et al., 2018), and further tested this using Egger’s linear regression method (Egger et al., 1997). Sensitivity analyses were conducted to assess the robustness of the results; for multi-arm randomized controlled trials that required pooled analysis, the impact of the study’s inclusion or exclusion on the overall results was further evaluated by repeating the analysis after excluding the study in question.

#### 2.3.4. Analysis of Moderating Factors

To investigate potential sources of heterogeneity, subgroup analyses and meta-regression analyses were conducted. First, this study performed subgroup analyses for each categorical variable to examine the impact of different categorical variables on intervention outcomes. Since subgroup analyses overlook potential correlations and interactions between variables (Carrero et al., 2019), this study subsequently employed meta-regression to explore factors influencing intervention outcomes. The Cochrane Handbook generally recommends including at least 10 studies for each level of analysis (Cumpston et al., 2019); R. Fu et al. (2011) suggest that 6 to 10 studies can serve as an empirical reference point for considering meta-regression, and that each subgroup of categorical variables should include at least 4 studies (R. Fu et al., 2011). Given the limited number of studies included in this research and the inclusion of multiple moderator variables in the model, the results of the correlation analysis are primarily intended to provide exploratory insights and should not be used to draw definitive conclusions.

Based on the characteristics of the included studies, this study preliminarily identified variables such as age, target population, region, intervention site, intervention duration, intervention format, trial type, and project type as moderating factors. Furthermore, following the dual-factor model, the outcome measures were categorized into two dimensions: positive mental health and negative psychological symptoms. Due to insufficient numbers of studies in the regions, trial types, and certain outcome dimensions—which failed to meet the minimum study count requirements for subsequent analyses—these were not included in the formal subgroup analyses or meta-regression models. Ultimately, a subgroup analysis was conducted for the positive mental health dimension, and the meta-regression analysis of the overall mental health effect included five variables: age, participant type, intervention format, intervention location, and intervention duration.

## 3. Results

### 3.1. Inclusion and Coding of Literature

**Figure 1:**
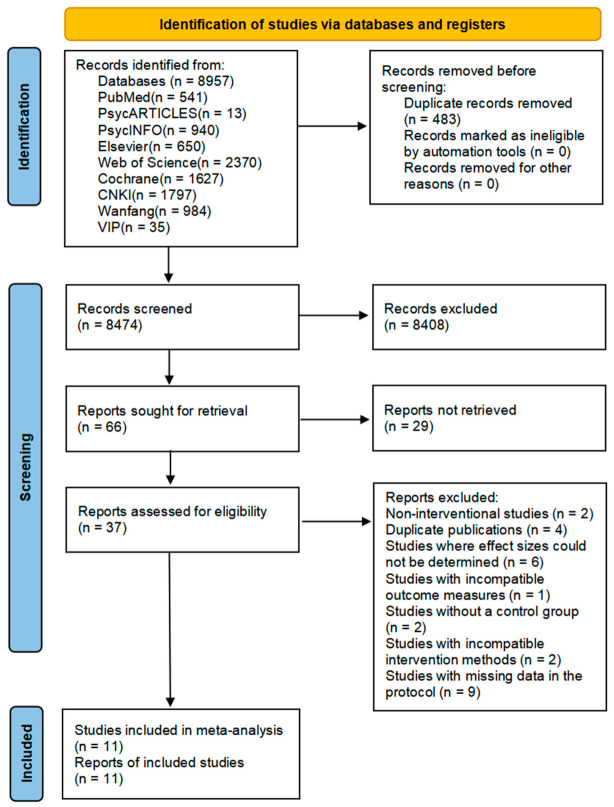
Flowchart of literature retrieval and screening.

**Figure 2:**
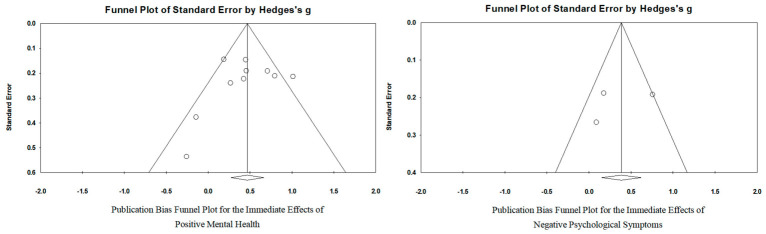
Publication bias funnel plots.

The literature screening process is shown in Figure 1. A total of 11 studies meeting the criteria were included (Fenner et al., 2016; H. Y. Jiang, 2022; Moola et al., 2017; Ornes et al., 2005; Pollock et al., 2023; Sacher et al., 2010; Sepúlveda et al., 2024; Vos et al., 2012; Young et al., 2019; F. Z. Zhang, 2024; X. Zhang et al., 2025), comprising 9 English-language and 2 Chinese-language studies, representing 14 research projects with a total sample size of 1160 participants. Three studies were conducted in China, involving 388 participants, while eight studies were conducted abroad, involving 772 participants. Five studies included follow-up, all with a duration of 6 months or longer; two of these studies also included follow-up periods of less than 6 months. Participants included both special populations and the general population: 529 participants from special populations (6 studies), including 13 children with cystic fibrosis (1 study) and 516 obese participants (5 studies), and 631 participants from the general population (5 studies). Regarding assessment tools, 10 were classified as low risk, 1 as high risk, and 0 as unclear risk; for quality assessment, 4 were classified as low risk of bias, 3 as unclear risk of bias, and 4 as high risk of bias. The overall risk-of-bias judgements are presented in Table 1, with detailed domain-level coding provided in Table S1. The publication-bias assessment was further visualized using funnel plots (Figure 2).

### 3.2. The Effectiveness of Family Physical Activity Interventions

The effects of the intervention are categorized into positive mental health outcomes and negative mental health symptoms. To avoid confounding effects from other variables during follow-up, these results represent the immediate effects of the intervention. Specific intervention outcomes are shown in Table 2.

**Figure 3:**
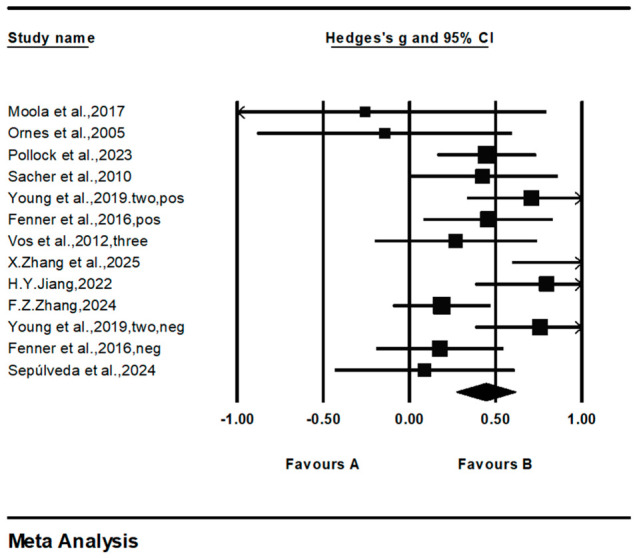
Forest diagram of overall mental health dimensions. “Favours A” and “Favours B” represent the negative and positive directions of the effect size, respectively. In this meta-analysis, positive values (favouring B) indicate a beneficial effect of the intervention compared with the control condition. Date from: Fenner et al. (2016); H. Y. Jiang (2022); Moola et al. (2017); Ornes et al. (2005); Pollock et al. (2023); Sacher et al. (2010); Sepúlveda et al. (2024); Vos et al. (2012); Young et al. (2019); F. Z. Zhang (2024); X. Zhang et al. (2025).

Among the included studies, a total of 13 reported on the overall mental health dimension (Figure 3). As shown in Table 2, regarding the immediate effects of the intervention, the overall pooled effect size for family physical activity interventions included in this study was (*g* = 0.443, 95% CI: 0.272–0.614, *p* < 0.001), suggesting that it is generally associated with improved mental health in children and adolescents. Sensitivity analysis results showed that the pooled effect size did not fluctuate significantly after excluding individual studies, indicating that the conclusions of this study are robust. Heterogeneity results showed *Q* = 26.19*, *df* = 12, *I*^2^ = 54.17%, indicating moderate heterogeneity among the studies. The Egger’s test *p*-value for publication bias was 0.47, indicating no statistical evidence of significant publication bias. Given that the included programs were not entirely homogeneous in terms of family participation methods and program composition, and given the limited number of studies, the relevant results should still be interpreted with caution in light of this heterogeneity.

**Figure 4:**
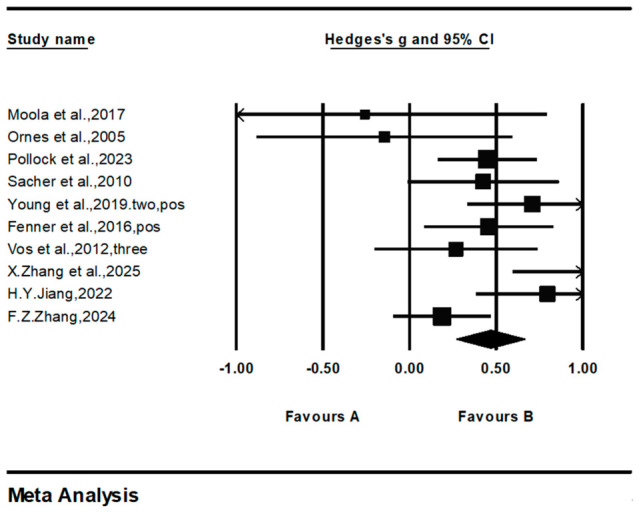
Forest diagram of positive mental health dimensions. Date from: Fenner et al. (2016); H. Y. Jiang (2022); Moola et al. (2017); Ornes et al. (2005); Pollock et al. (2023); Sacher et al. (2010); Vos et al. (2012); Young et al. (2019); F. Z. Zhang (2024); X. Zhang et al. (2025).

For the positive mental health dimension, 10 studies involving a total of 995 participants were included (Figure 4). The overall effect size was (*g* = 0.467, 95% CI: 0.271 to 0.663, *p* < 0.001), indicating that family physical activity has a significant positive effect on improving adolescents’ positive mental health; heterogeneity results showed *Q* = 19.56*, *df* = 9, *I*^2^ = 53.98%, suggesting a moderate degree of heterogeneity among the studies.

**Figure 5:**
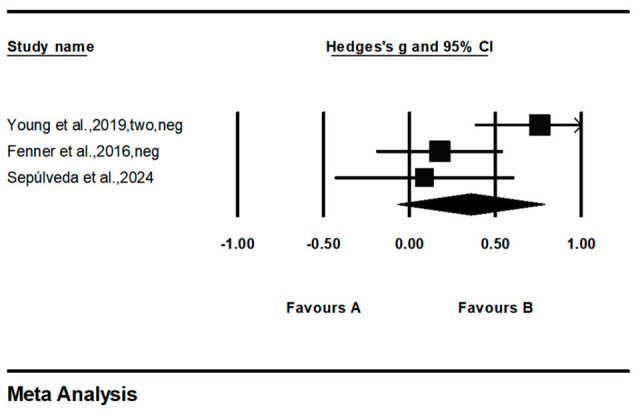
Forest diagram of negative psychological symptoms. Date from: Fenner et al. (2016); Sepúlveda et al. (2024); Young et al. (2019).

In the dimension of negative psychological symptoms, three studies involving a total of 336 participants were included (Figure 5). The pooled effect size was (*g* = 0.358, 95% CI = −0.067 to 0.784, *p* > 0.05), with the 95% confidence interval crossing zero, indicating that the current evidence is insufficient to support a significant improvement in negative psychological symptoms among children and adolescents through family physical activity. Heterogeneity results showed *I*^2^ = 68.05%, but the *Q* and *df* values did not reach statistical significance.

### 3.3. Subgroup Analysis of Home-Based Physical Activity Interventions

Due to the limited number of included studies, a subgroup analysis was conducted only on the immediate effects of interventions targeting positive mental health. Based on the moderating variables influencing the effectiveness of family physical activity interventions and the characteristics of the included studies, the following subgroups were identified: (1) Target population: general population vs. special populations; (2) Age of participants: 12 years or younger vs. 12 years or older; (3) Intervention format: joint participation vs. supportive assistance; (4) Primary intervention setting: home-based vs. non-home-based; (5) Intervention duration: within three months vs. beyond three months; (6) Project type: multi-component projects vs. non-multi-component projects.

The positive effects of family physical activity interventions on positive mental health were generally consistent across different subgroups. Subgroup analysis results showed that family physical activity interventions generally had a positive effect on the immediate outcomes of positive mental health across various subgroups, including intervention participants, intervention format, age, intervention location, intervention duration, and program type. Significant positive effects were observed in all subgroups, including the general population, special populations, co-participation, assisted support, children aged 12 and under, children aged 12 and over, non-home-based interventions, intervention durations of ≤3 months and >3 months, and multi-component programs. However, the effects in the home-based subgroup and the non-multi-component program subgroup did not reach statistical significance. The results of the tests for differences between subgroups were not statistically significant (all *p*-values were greater than 0.05), indicating that, within the scope of the evidence included in this study, no between-group differences were observed among the aforementioned variables. The I^2^ values for the subgroups of co-participation, home-based interventions, intervention duration ≤ 3 months, and multi-component programs were all below 50%, suggesting relatively good consistency of results within these subgroups. Specific results are shown in Table 3.

### 3.4. Meta-Regression Analysis of the Effects of Mental Health Early Intervention

The results of the heterogeneity test for the overall mental health effect showed that the *Q* statistic reached statistical significance, with *I*^2^ = 54.17%, indicating moderate heterogeneity among the included studies. Given that the relatively limited number of studies on positive mental health and negative psychological symptoms somewhat restricts the meta-regression’s ability to detect true moderating effects, a meta-regression analysis was conducted solely on the overall immediate mental health effects. To further explore potential sources of heterogeneity among studies, this study employed a random-effects meta-regression model to conduct an exploratory analysis of moderating factors for family physical activity interventions. Due to the limited number of studies, five variables—age, target population, intervention format, intervention location, and intervention duration—were selected as moderating variables. Model 1 included the age variable; Model 2 included the type of participants; and Model 3 included intervention characteristics: intervention format, intervention location, and intervention duration.

The results show that Model 3 explains approximately 22% of the between-study heterogeneity (*R*^2^analog = 0.22); however, the overall test did not reach statistical significance (*Q* = 7.53, *df* = 5, *p* = 0.1841). This suggests that the meta-regression failed to identify significant moderating factors at the overall model level, and the included moderating variables were insufficient to reliably explain the between-study differences in intervention effects. It should be noted that, given the relatively limited number of studies included in this research and the inclusion of multiple moderator variables in the model, the meta-regression results are primarily intended to provide exploratory insights and should not be interpreted as confirmatory inferences. Although individual regression coefficients show certain trends, these results should not be interpreted as definitive. Overall, the multivariate meta-regression in this study has not identified any significant moderator factors supported by the overall model test. See Table 4 for detailed meta-regression model specifications and corresponding statistical results.

## 4. Discussion

Based on evidence from controlled trials, this study systematically examined the effects of home-based physical activity interventions on the mental health of children and adolescents aged 5 to 19. Overall, family involvement may provide a more conducive setting for implementing physical activity interventions among children and adolescents and plays a positive role in promoting mental health.

### 4.1. The Immediate Effects of Family Physical Activity on Mental Health

Based on evidence from controlled trials, this study found that family physical activity interventions are generally associated with improved mental health among children and adolescents aged 5–19 years. Given the differences among the included programs in terms of family participation methods, program composition, and additional components, this overall effect is best interpreted as a composite estimate of various heterogeneous family physical activity approaches. Subgroup analysis further revealed that the positive effect of family physical activity on positive mental health reached statistical significance (*g* = 0.467, 95% CI: 0.271–0.663, *p* < 0.001), whereas the effect on reducing negative psychological symptoms did not reach statistical significance (*p* > 0.05). This finding is consistent with the conclusions of a meta-analysis by Andermo et al. (2020) on physical activity interventions for general child and adolescent populations: while physical activity interventions help improve positive mental health, evidence for alleviating psychopathological symptoms is relatively insufficient (Andermo et al., 2020). Based on this, it can be inferred that the impact of family physical activity on the overall mental health of children and adolescents may primarily manifest as an enhancement of positive psychological resources, rather than a direct reversal of existing psychological symptoms.

### 4.2. Moderating Factors and Implications for Intervention Effects

Although differences between subgroups did not reach statistical significance, the effect size for non-home-based interventions (*g* = 0.55, 95% CI: 0.356 to 0.749) was higher than that for home-based interventions (*g* = 0.26, 95% CI: −0.192 to 0.703), and meta-regression analysis showed a marginally negative trend for home-based interventions (*b* = −0.94, 95% CI: −1.89 to 0.003, *p* = 0.051). These findings suggest that there are certain differences in the distribution of effect sizes between home-based and non-home-based settings. In light of existing research, these differences may be related to variations in program organization, forms of interactive support, and implementation conditions. However, given that the differences between groups were not statistically significant and the number of studies is limited, the current evidence is insufficient to determine the consistent mechanisms underlying the influence of setting factors. The relevant literature suggests that some non-home-based programs may be more conducive to sustained participation and positive experiences due to clearer activity organization, more substantial face-to-face interactive support, and relatively stable implementation conditions; however, this explanation requires further validation through additional research (Moss & Gu, 2022). The effect size for interventions lasting three months or less was higher than that for interventions lasting longer than three months (*g* = 0.54 vs. *g* = 0.36). Only five studies with follow-up data were included, all of which had follow-up periods of six months or longer; two of these also reported follow-up within six months, indicating a relative lack of long-term evidence. This suggests that the psychological benefits of family physical activity may be more readily apparent in the early stages of intervention. Future research should focus more on how to promote the long-term maintenance of the benefits of family physical activity interventions.

Significant positive effects were observed in both the general population and the subgroup of special populations, and the difference between the groups was not statistically significant (*g* = 0.45 vs. *g* = 0.49, *p* = 0.856), suggesting that home-based physical activity has a certain degree of cross-population applicability. However, universality does not equate to a one-size-fits-all approach. It is worth noting that variations in effect sizes across different demographic groups may be related to differences in sample characteristics and intervention design. In a study of children with cystic fibrosis, Moola et al. demonstrated that home-based physical activity interventions successfully increased moderate-to-vigorous physical activity levels (+6 min/day), but scores on the social dimension of quality of life showed a downward trend in the intervention group (effect size decreased from 80 to 73.33) (Moola et al., 2017). This result may reflect that, for children and adolescents facing specific disease management challenges, interventions focused on “overcoming barriers” and “self-management” may inadvertently reinforce perceptions of disease limitations and even have negative effects on mental health. Therefore, future studies should optimize intervention content based on the characteristics of different populations.

Further stratification by project type revealed that the multi-component project subgroup exhibited a significant positive effect (*g* = 0.55, 95% CI: 0.356–0.749), whereas the effect in the non-multi-component project subgroup was not statistically significant (*g* = 0.26, 95% CI: −0.192 to 0.703); however, the test for between-group differences did not reach statistical significance (*p* = 0.233). This suggests that, at this stage, it is not yet possible to conclude that the type of program exerts a consistent moderating effect. However, judging from the distribution of effect sizes and the composition of the programs, the differences observed across studies may not merely reflect variations in the intensity of effects among similar interventions; compared to programs focused solely on physical activity, multi-component interventions may also incorporate additional modules such as dietary guidance, behavior management, or lifestyle adjustments, thereby jointly influencing the mental health of children and adolescents through more complex pathways. Given that the above conclusions are still based on exploratory subgroup comparisons and that the tests for between-group differences did not reach statistical significance, the current evidence is insufficient to confirm which specific additional components and their mechanisms of action account for the relative advantages of multi-component programs. In other words, the inter-study variability observed not only manifests as statistically significant differences in effect sizes but may also reflect conceptual differences in the program structure and mechanisms of action among the included interventions. This further suggests that the overall effect observed in this study is best understood as a comprehensive estimate of family physical activity intervention programs with varying structures and mechanisms of action, rather than simply as the average effect of a single intervention model. Future studies should provide more detailed reports on intervention content to enhance comparability among different programs and improve the accuracy of result interpretation.

It should be noted that the subgroup analyses and moderation analyses described above are exploratory in nature. Due to limitations in the number of included studies and statistical power, their primary purpose is to describe differences in the distribution of effect sizes across studies with varying characteristics and to provide insights into the heterogeneity among studies; they should not be used to draw firm conclusions about moderation effects or to infer definitive mechanisms.

### 4.3. Limitations of the Study

The recommendations outlined above aim to strengthen the evidence base in this field; however, this study may have the following limitations: (1) The total number of studies included in the review was limited, particularly regarding the dimension of negative psychological symptoms, which restricted the estimation of the combined effect for this dimension. Furthermore, the study did not compare differences in intervention effects across different follow-up periods; the current conclusions primarily reflect the post-intervention effects of family physical activity interventions, and evidence regarding their long-term maintenance effects remains relatively limited. (2) This study included only 11 studies in the meta-regression analysis, yet the model incorporated five predictor variables. Given the small number of studies and the large number of predictor variables, there is a high risk of model overfitting. The overall meta-regression model did not reach statistical significance, making it impossible to identify stable and reliable key moderating factors based on this analysis. Therefore, current assessments of potential sources of heterogeneity remain largely exploratory in nature, and confirmatory inferences should not be drawn at this stage. (3) Furthermore, this study included only Chinese and English literature, which may introduce a certain degree of language bias. Future research could expand the language scope to enhance the comprehensiveness of the evidence. (4) The included studies comprised a number of multi-component programs; in addition to the core component of family physical activity, these interventions incorporated supplementary modules such as dietary guidance, behavior management, or lifestyle modifications. Current evidence is insufficient to further distinguish the independent contributions of the core component of family physical activity from these additional modules. Therefore, the overall effect observed in this study should be interpreted as a composite estimate of heterogeneous family physical activity interventions rather than simply attributed to the core component of family physical activity itself. This, to some extent, limits the attributability of the results and the explanation of underlying mechanisms. Future studies should report intervention characteristics and sample background information in a more standardized and detailed manner, and provide more operational definitions of modules such as exercise components and forms of family participation, in order to improve the accuracy of result interpretation and provide more robust evidence for the development of implementable intervention protocols.

## Figures and Tables

**Table 1:** Basic characteristics of the literature included in the study on the impact of family sports on children and adolescents’ mental health.

Author (Year)	Research Type	Sample Size	Measurement Time Point	Sample Type	Age	Intervention Type	Project Type	Intervention Site	Intervention Frequency	Intervention Cycle	Outcome Variable	Measuring Tools	Evaluation Results	Research Quality
Moola et al. (2017)	RCT	13 (7, 6)	Pre-test/Post-test	Vulnerable groups	13.9 ± 1.5	Assist with	non-multi-component projects	At home	Once/two weeks, 90 min	8 weeks	Negative psychological symptoms	PedsQL	Low risk	unclear risk of bias
Ornes et al. (2005)	Non-RCT	36 (27, 9)	Pre-test/Post-test	General groups	10.1 ± 1.5	Participate together	non-multi-component projects	At home	Three times/week, duration per session not reported	6 months	Positive Mental Health	SWLS	Low risk	unclear risk of bias
Pollock et al. (2023)	RCT	192 (98, 94)	Pre-test/Post-test	General groups	8.4 ± 1.9	Participate together	multi-component projects	Not at home	Once/week, plus weekly homework assignments	9 weeks	Positive Mental Health	DESSA, KINDL-R	Low risk	Low risk of bias
Sacher et al. (2010)	RCT	116 (60, 56)	Pre-test/Post-test, Tracked for more than 6 months	Vulnerable groups	10.3 ± 1.3	Assist with	multi-component projects	Not at home	Twice/week	6 months	Positive Mental Health	Harter Self-Perception Profile	Low risk	High risk of bias
Young et al. (2019)	RCT	115 (57, 58)	Pre-test/Post-test, Tracked for more than 6 months	General groups	7.7 ± 1.8	Participate together	multi-component projects	Not at home	Once/week, 90 min, plus family challenge assignments	8 weeks	Positive Mental Health, Negative psychological symptoms	DESSA, SDQ	Low risk	Low risk of bias
Fenner et al. (2016)	Non-RCT	56 (Self-comparison)	Pre-test/Post-test, Tracked for more than 6 months	Vulnerable groups	13.9 ± 1.5	Assist with	multi-component projects	Not at home	Twice/week, two hours	8 weeks	Positive Mental Health, Negative psychological symptoms	SMFQ, PedsQL	Low risk	High risk of bias
Vos et al. (2012)	RCT	79 (40, 39)	Pre-test/Post-test, Tracked for more than 6 months	Vulnerable groups	13.2 ± 2.0	Assist with	multi-component projects	Not at home	Once/two weeks, two hours	3 months	Positive Mental Health	DISABKIDS	Low risk	Low risk of bias
Sepúlveda et al. (2024)	RCT	165 (62, 52, 51)	Pre-test/Post-test, Tracked for more than 6 months	Vulnerable groups	10.3 ± 1.4	Assist with	multi-component projects	Not at home	Once/two weeks, two hours	6 months	Negative psychological symptoms	CDI, SCAS	Low risk	unclear risk of bias
X. Zhang et al. (2025)	RCT	100 (50, 50)	Pre-test/Post-test	Vulnerable groups	14.0 ± 1.0	Assist with	multi-component projects	Not at home	Three times/week, one hour	12 weeks	Positive Mental Health	Rosenberg	Low risk	Low risk of bias
H. Y. Jiang (2022)	RCT	96 (49, 47)	Pre-test/Post-test	General groups	16.5 ± 1.5	Participate together	non-multi-component projects	At home	3–4 times/week, 15–30 min	4 months	Positive Mental Health	Middle School Student School Adjustment Scale	High risk	High risk of bias
F. Z. Zhang (2024)	RCT	192 (96, 96)	Pre-test/Post-test	General groups	13.5 ± 1.0	Assist with	non-multi-component projects	At home	At least 30 min daily on weekends; at least 3/4 times/week for 30 min or more during summer break	17 weeks	Negative psychological symptoms	Survey Questionnaire on the Current Status of Health Behaviors Among Junior High School Students (expert-rated, α = 0.889, test–retest r ≈ 0.83)	Low risk	High risk of bias

**Table 2:** Family-based physical intervention: Effect size, heterogeneity test, and publication bias test.

Outcome Variable	*k*	*N*	*g* (95%CI)	Sensitivity Analysis	Heterogeneity Test			Publication Bias Test				
				*g* (95%CI)	*Q*_w_	*df*	*I* ^2^	*N_fs_*	Egger’s Intercept	*SE*	(95%CI)	*p*
Mental health	13	1160	0.443 *** (0.272, 0.614)	No outliers	26.19 *	12	54.17	169	−1.11	1.50	(−4.40, 2.18)	0.47
Positive	10	995	0.467 *** (0.271, 0.663)	No outliers	19.56 *	9	53.98	107	−0.834	1.59	(−4.49, 2.82)	0.61
Negative	3	336	0.358 (−0.067, 0.784)	No outliers	6.26	2	68.05	5	−4.42	8.75	(−115.55, 106.71)	0.70

Note: * *p* < 0.05, *** *p* < 0.001.

**Table 3:** Subgroup analysis results of positive mental health.

Subgroup	*k*	*g* 95%CI	*Z*	*Q*	*I*^2^ (%)	*p*
**Subject type**						
Vulnerable groups	5	0.45 (0.175, 0.717)	3.255 **	10.29 *	61.12	0.856
General groups	5	0.49 (0.163, 0.807)	2.949 **	8.87 *	54.89	
**Intervention type**						
Assist with	4	0.54 (0.249, 0.828)	3.645 ***	5.92	49.35	0.555
Participate together	6	0.42 (0.137, 0.697)	2.921 **	12.36 *	59.53	
**Age**						
12 years or younger	6	0.48 (0.172, 0.790)	3.05 **	15.28 **	67.28	0.913
12 years and older	4	0.46 (0.214, 0.703)	3.67 ***	4.27	61.19	
**Intervention site**						
Not at home	6	0.55 (0.356, 0.749)	5.51 ***	7.85	36.26	0.233
At home	4	0.26 (−0.192, 0.703)	1.12	8.61 *	65.15	
**Intervention cycle**						
Within 3 months	6	0.54 (0.296, 0.779)	4.36 ***	9.78	48.97	0.412
More than 3 months	4	0.36 (0.019, 0.705)	2.07 *	7.57	60.39	
**Project type**						
multi-component projects	6	0.55 (0.356, 0.749)	5.51 ***	7.85	36.26	0.233
non-multi-component projects	4	0.26 (−0.192, 0.703)	1.12	8.61 *	65.15	

Note: * *p* < 0.05, ** *p* < 0.01, *** *p* < 0.001.

**Table 4:** Meta-regression analysis of the effects of moderating variables on family sports intervention.

Moderating Variables	Model 1		Model 2		Model 3	
	*b* (95%CI)	*SE*	*b* (95%CI)	*SE*	*b* (95%CI)	*SE*
**Age**						
12 years or younger	0.01 (−0.34, 0.37)	0.183	−0.04 (−0.44, 0.37)	0.206	−0.67 (−1.32, −0.02)	0.332
12 years and older	—	—	—	—	—	—
**Subject type**						
Vulnerable groups	—	—	−0.13 (−0.3, 0.27)	0.205	−0.22 (−0.97, 0.52)	0.379
General groups	—	—	—	—	—	—
**Intervention type**						
Assist with	—	—	—	—	−0.56 (−1.29, 0.17)	0.372
Participate together	—	—	—	—	—	—
**Intervention site**						
At home	—	—	—	—	−0.95 (−1.92, 0.01)	0.492
Not at home	—	—	—	—	—	—
**Intervention cycle**						
More than 3 months	—	—	—	—	0.42 (−0.24, 1.07)	0.334
Within 3 months	—	—	—	—	—	—
*R*^2^	0.00		0.00		0.22	
*Q* (*df*)	0.01 (1)		0.42 (2)		7.53 (5)	

## Data Availability

All data analyzed during this study are included in the published article and its Supplementary Information Files.

## Supplementary Materials

The following supporting information can be downloaded at: https://www.mdpi.com/article/10.3390/bs16050776/s1, Table S1: PRISMA 2020 checklist (Page et al., 2021); File S1: Full Search Strategies.
