# Supplementary material for: The Effects of Family Sports on Mental Health in Children and Adolescents: A Meta-Analysis
Source: Behav Sci (Basel). 2026 May 14;16(5):776. doi: 10.3390/bs16050776 (PMC13203365; doi:10.3390/bs16050776)
Supplement: Supplementary file 1 [file behavsci-16-00776-s001.zip › Supplementary S2. Full Search Strategies.pdf]

PubMed=541
(("Motor Activity"[MeSH Terms] OR "Exercise/psychology"[MeSH Terms] OR "physical activity"[Title/Abstract]) AND ("Family"[MeSH Terms] OR "Parents"[MeSH Terms] OR "family-based"[Title/Abstract] OR "family intervention*"[Title/Abstract] OR "family-based interventions"[Title/Abstract] OR "parent-child interaction"[Title/Abstract] OR "family sports activities"[Title/Abstract]) AND ("Adolescent"[MeSH Terms] OR "Child"[MeSH Terms] OR adolescent*[Title/Abstract] OR youth[Title/Abstract]) AND ("Mental Health"[MeSH Terms] OR "Depression"[MeSH Terms] OR "Anxiety"[MeSH Terms] OR "mental health"[Title/Abstract] OR psychological[Title/Abstract] OR "psychosocial outcomes"[Title/Abstract] OR "mental well-being"[Title/Abstract] OR "psychological distress"[Title/Abstract]))

Web of Science new=2370
"Motor Activity" OR "Exercise" OR "physical activity" (Topic) and "Family" OR "Parents" OR "family-based" OR "family intervention" OR "family-based interventions" OR "parent-child interaction" OR "family sports activities" (Topic) and "Adolescent" OR "Child*" OR "adolescent*" OR "youth" (Topic) and "Mental Health" OR "Depression" OR "Anxiety" OR "mental health" OR "psychological" OR "psychosocial outcomes" OR "mental well-being" OR "psychological distress" (Topic)

Elsevier=650
("Motor Activity" OR Exercise OR "physical activity") AND (Family OR Parents OR "family-based" OR "family intervention*" OR "family-based interventions" OR "parent-child interaction" OR "family sports activities") AND (Adolescent OR Child* OR adolescent* OR youth) AND ("Mental Health" OR Depression OR Anxiety OR "mental

health" OR psychological OR "psychosocial outcomes" OR "mental well-being" OR "psychological distress")

Cochrane=1627
"Motor Activity" OR "Exercise" OR "physical activity" in Title Abstract Keyword AND "Family" OR "Parents" OR "family-based" OR "family intervention" OR "family-based interventions" OR "parent-child interaction" OR "family sports activities" in Title Abstract Keyword AND "Adolescent" OR "Child*" OR "adolescent*" OR "youth" in Title Abstract Keyword AND "Mental Health" OR "Depression" OR "Anxiety" OR "mental health" OR "psychological" OR "psychosocial outcomes" OR "mental well-being" OR "psychological distress" in Title Abstract Keyword - (Word variations have been searched)

PsycARTICLES=13、PsycINFO=940
("Motor Activity" OR "Exercise" OR "physical activity") AND XB ("Family" OR "Parents" OR "family-based" OR "family intervention" OR "family-based interventions" OR "parent-child interaction" OR "family sports activities") AND XB ("Adolescent" OR "Child*" OR "adolescent*" OR "youth") AND XB ("Mental Health" OR "Depression" OR "Anxiety" OR "mental health" OR "psychological" OR "psychosocial outcomes" OR "mental well-being" OR "psychological distress")

中国知网=1797
(篇关摘: "体育" + "体育活动" + "体育锻炼" + "身体活动" + "运动" + "健身" + "运动干预"(精确)) AND (篇关摘: "家庭" + "亲子" + "父母" + "家长" + "家庭教育" + "亲子互动" + "家庭支持" + "家长参与" + "居家"(精确)) AND (篇关摘: "青少年" + "中学生" + "初中生" + "高中生" + "未成年人"(精确)) AND (篇关摘: "心理健康" + "情绪调节" + "社会适应" + "自尊" + "焦虑" + "抑郁" + "心理卫生" + "自信" + "同伴关系" + "冲突" + "情绪" + "自杀"(精确))

万方=984

(主题: (体育) or 题名或关键词:(体育 or 体育活动 or 体育锻炼 or 身体活动 or 运动 or 健身 or 运动干预)) and (主题: (家庭) or 题名或关键词:(居家 or 亲子 or 父母 or 家长 or 家庭教育 or 亲子互动 or 家庭支持 or 家长参与)) AND (主题: (儿童青少年) or 题名或关键词:(青少年 or 中学生 or 初中生 or 高中生 or 未成年人)) AND (主题: (心理健康) or 题名或关键词:(情绪调节 or 社会适应 or 自尊 or 焦虑 or 抑郁 or 心理卫生 or 自信 or 同伴关系 or 冲突 or 情绪 or 自杀))

VIP Database

(题名或关键词 = 体育 OR 体育活动 OR 体育锻炼 OR 身体活动 OR 运动 OR 健身 OR 运动干预) AND (题名或关键词 = 家庭 OR 亲子 OR 父母 OR 家长 OR 家庭教育 OR 亲子互动 OR 家庭支持 OR 家长参与 OR 居家) AND (题名或关键词 = 青少年 OR 中学生 OR 初中生 OR 高中生 OR 未成年人) AND (题名或关键词 = 心理健康 OR 情绪调节 OR 社会适应 OR 自尊 OR 焦虑 OR 抑郁 OR 心理卫生 OR 自信 OR 同伴关系 OR 冲突 OR 情绪 OR 自杀)
